# Elevation‐Driven Morphological Variation in *Dianthus virgineus* L. s.l. in the Southern Apennine: A Functional Perspective

**DOI:** 10.1002/ece3.73898

**Published:** 2026-07-08

**Authors:** Simone Rovito, Domenico Amantea, Nicodemo Giuseppe Passalacqua, Liliana Bernardo, Domenico Gargano

**Affiliations:** ^1^ Department of Biology, Ecology and Earth Sciences University of Calabria Rende Italy

**Keywords:** *dianthus*, fluctuating asymmetry, functional traits, phenotypic canalization, plant adaptation

## Abstract

Traditional morphological frameworks within the *Dianthus virgineus* complex have recognized two closely related taxa, *D. longicaulis* and *D. brachycalyx*, distributed along elevational gradients in the central and southern Apennines. However, recent genomic evidence has questioned their taxonomic independence, suggesting that morphological differentiation may reflect environmentally structured variation rather than distinct evolutionary lineages. Here, we adopted an integrative trait‐based approach to evaluate whether phenotypic discontinuities occur between low‐ and high‐elevation populations of *D. virgineus* in the southern Apennines. We analyzed morphological traits, functional traits, and fluctuating asymmetry across 12 populations spanning a broad elevation range (500–2200 m a.s.l.), treating elevation as a proxy for ecological variation. Morphological differentiation was significant but continuous along the elevational gradient, with no evidence of discrete phenotypic clusters corresponding to traditionally recognized taxa. Functional traits showed coordinated shifts consistent with increasing stress tolerance at higher elevations, and multivariate analyses revealed significant congruence between morphological and functional trait spaces. Patterns of fluctuating asymmetry decreased with elevation, suggesting enhanced developmental stability under persistent environmental constraints. Together, these results indicate that the phenotypic combinations historically used to delimit *D. longicaulis* and *D. brachycalyx* are best interpreted as environmentally structured morphotypes within *D. virgineus* , rather than taxonomically independent entities. Our findings highlight how integrative analyses combining morphology, functional ecology, and developmental stability can clarify the ecological and evolutionary significance of phenotypic variation in polymorphic plant complexes and help prevent taxonomic oversplitting.

## Graphical abstract

Our study on *Dianthus virgineus* uses morphology, functional traits, and fluctuating asymmetry to explore elevational phenotypic variation. We found continuous, environmentally‐driven variation, suggesting local adaptation over distinct species. This work offers a framework to help in the interpretation of polymorphic plant complexes and prevent taxonomic oversplitting.

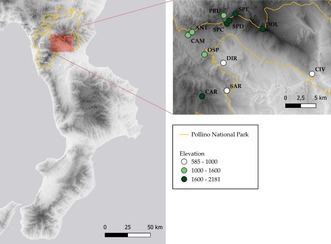

## 1 Introduction

Understanding the extent to which morphological variation reflects evolutionary divergence rather than environmentally associated phenotypic variation, including phenotypic plasticity and/or local adaptation, remains a central challenge in plant systematics. In many taxonomic complexes, morphological differentiation occurs along environmental gradients, potentially generating geographically structured morphotypes that may be erroneously interpreted as distinct taxa when genetic cohesion is maintained (De Queiroz 2007; Sites Jr. and Marshall 2004). Integrative approaches combining morphology with ecological and functional trait data are therefore essential for disentangling environmentally induced variation from lineage divergence, particularly in polymorphic plant groups distributed across heterogeneous landscapes.

The genus *Dianthus* L. (Caryophyllaceae), which includes pinks and carnations, has undergone rapid diversification, particularly since the Plio–Holocene epochs, resulting in high species richness and marked taxonomic complexity in the Mediterranean Basin (Valente et al. 2010; Fassou et al. 2022). Several evolutionary and ecological factors have contributed to this diversification, including polyploidy (Balao et al. 2010; Terlevic´ et al. 2022; Franzoni, Astuti, Bacchetta, et al. 2024), recombination among diverging lineages (Fior et al. 2025), variation along environmental gradients, and adaptation to heterogeneous edaphic conditions (Bacchetta et al. 2010; Hamzaog˘lu et al. 2015; Cahenzli et al. 2018; López‐Jurado et al. 2019). As a consequence, many *Dianthus* lineages comprise polymorphic populations showing high levels of intraspecific variation, often resulting in blurred phenotypic boundaries and taxonomic uncertainty (Barina et al. 2020; Hardion et al. 2020; Castro et al. 2022; Rocha et al. 2026).

In several species, phenotypic differentiation occurs along altitudinal gradients, where gradual environmental variation is associated with changes in plant morphology (Cordell et al. 1998; Byars et al. 2007). However, the taxonomic interpretation of such patterns is frequently complicated by phenotypic continuity among populations and by the difficulty of disentangling environmentally structured variation from genetically based differentiation (Crespi et al. 2007; Franzoni, Astuti, Bartolucci, et al. 2024).

For instance, recent studies interpret the morphological variation in the genus *Dianthus* as a resilient network of morpho‐environmental strategies, contributing to continuity between species and making their classification uncertain (Rocha et al. 2026).

The *Dianthus virgineus* *L. complex* (formerly treated under the name *D. sylvestris* Wulfen; Domina, Astuti, Bacchetta, et al. 2021; Domina, Astuti, Barone, et al. 2021) provides a well‐known example of this complexity. It includes morphologically heterogeneous populations distributed throughout Central and Western Europe, with major centers of diversity in the Balkans and the Apennines (Bacchetta et al. 2010; Terlevic´ et al. 2022; Franzoni, Astuti, Bacchetta, et al. 2024). Recent morphological and genomic studies (Gargano et al. 2023; Luqman et al. 2023) support the existence of three main evolutionary lineages within the complex: one occurring in the Western Alps and Apennines ( *D. virgineus* s.s.), a second in the Central Alps (*D. inodorus* L.), and a third in the Eastern Alps and the Balkans ( *D. sylvestris* ). Within *D. virgineus* sensu lato, phenotypic variation along elevational gradients has often led to the description of local morphotypes as distinct taxa (Bacchetta et al. 2010), although recent evidence suggests that many of these differences reflect continuous, environmentally structured variation rather than discrete evolutionary lineages (Franzoni et al. 2023; Franzoni, Astuti, Bacchetta, et al. 2024).

In the Southern Apennines, populations traditionally referred to *D. longicaulis* Ten. and *D. brachycalyx* have been interpreted as the result of ecological vicariance along an elevational gradient (Bacchetta et al. 2010). Nevertheless, recent genomic analyses failed to support their separation, indicating that both entities fall within *D. virgineus* sensu lato (Franzoni, Astuti, Bacchetta, et al. 2024; Franzoni, Astuti, Bartolucci, et al. 2024). This system, therefore, represents an ideal case study for investigating how morphological variation is structured along environmental gradients and how such variation may contribute to taxonomic oversplitting.

Understanding how phenotypic variation is distributed among populations and across environmental gradients is central to both evolutionary ecology and systematics.

Functional traits—morphological, physiological, or phenological characteristics measurable at the individual level—summarize key aspects of plant ecological strategies and their relationships with environmental conditions (Weiher et al. 1999; Violle et al. 2007; Díaz et al. 2015). Trait–environment associations are particularly informative in plants, where persistence depends on the ability to cope with local ecological constraints (Grime and Pierce 2012; Díaz et al. 2015). Trait‐based approaches emphasize the use of standardized, functionally meaningful variables and harmonized measurement protocols (Weiher et al. 1999; Cornelissen et al. 2003; Kattge et al. 2011; Pérez‐Harguindeguy et al. 2013), allowing more consistent comparisons across studies and taxa than traditional ad hoc morphometric character sets. Coordinated shifts in traits such as plant height, leaf size, and resource‐use strategies along environmental gradients have been widely documented (Lavorel and Garnier 2002; McGill et al. 2006; Messier et al. 2010; Krishna et al. 2021) and are increasingly integrated into systematic investigations (Flores et al. 2014; Schneider et al. 2019).

Elevational gradients integrate multiple ecological factors—including temperature, precipitation, seasonality, and soil conditions—that can influence plant phenotype (McGill et al. 2006; Krishna et al. 2021). Along such gradients, functional traits often show predictable shifts, such as reduced leaf size and increased structural investment at higher elevations (Anthelme and Dangles 2012; Michalet et al. 2014), compared with larger and thinner leaves in lowland environments (Grime 1994). Although frequently interpreted as adaptive responses, these patterns may also reflect environmentally structured variation arising from the interaction between developmental processes and ecological constraints.

In this context, phenotypic variation may also be associated with differences in developmental stability. Fluctuating asymmetry (FA), defined as small random deviations from perfect bilateral symmetry, has been widely used as an indicator of developmental instability potentially associated with genetic or environmental stress (Palmer and Strobecke 1986; Palmer 1994; Tomkins and Kotiaho 2002; Réale and Roff 2003; Abeli et al. 2016; Perri et al. 2024). When interpreted cautiously, FA can provide complementary insight into how phenotypic variability is expressed across populations experiencing different ecological conditions (Debat and David 2001; Takahashi 2019).

Here, we investigate phenotypic variation in populations of *D. virgineus* distributed along a broad elevational gradient in the Southern Apennines, where low‐ and high‐elevation morphotypes have traditionally been assigned to *D. longicaulis* and *D. brachycalyx*. By integrating morphological traits used in traditional taxonomy with functional plant traits and measures of FA, we aim to clarify whether phenotypic differentiation along the gradient reflects discrete taxonomic entities or environmentally structured variation within a single lineage. Specifically, we aim to:

- Quantify morphological and functional variation among populations along the elevational gradient;
- Assess the degree of congruence between morphological and functional differentiation using multivariate analyses;
- Evaluate relationships between elevation and trait expression, treating elevation as a proxy for multiple ecological drivers; and
- Discuss the taxonomic implications of the observed phenotypic patterns in light of recent genomic evidence, with particular reference to the distinction between *D. longicaulis* and *D. brachycalyx*.

Through this integrative approach, we aim to contribute to the ongoing discussion on species delimitation within the *D. virgineus* complex by illustrating how environmentally structured phenotypic variation may contribute to taxonomic oversplitting.

## 2 Materials and Methods

### 2.1 Study System and Data Collection

Traditional taxonomic frameworks (Bacchetta et al. 2010; Brullo and Guarino 2017) have attributed the *D. virgineus* populations occurring in the study area to two taxa: *D. longicaulis* and *D. brachycalyx*. The former refers to a central‐western Mediterranean taxon occurring up to approximately 1500 m in various rocky habitats, including open pastures, forest edges, and partially wooded patches. It is characterized by long, branched stems bearing a varying amount of terminal, solitary flowers with an elongated calyx (Bacchetta et al. 2010; Brullo and Guarino 2017). In contrast, *D. brachycalyx* has been described as endemic to the central and southern Italian Peninsula, where it occurs in open high‐mountain environments (above 1500–1800 m a.s.l.). Its distinguishing features include a densely caespitose habit, short and weakly branched stems, and terminal flowers—solitary or few in number—bearing a short calyx. The present study was specifically focused on these two traditionally recognized southern Apennine elevational morphotypes, whose taxonomic distinction has been questioned by recent genomic evidence, rather than on a floristic survey of all Dianthus taxa occurring in the area or on comparative morphometric revision of the entire *D. virgineus* complex.

However, based on recent systematic achievements (Gargano et al. 2023; Luqman et al. 2023; Franzoni, Astuti, Bacchetta, et al. 2024; Franzoni, Astuti, Bartolucci, et al. 2024), the two phenotypes are currently treated as different variants of the species *D. virgineus* . Accordingly, we refer to all studied populations as *D. virgineus* throughout this paper.

**FIGURE 1:**
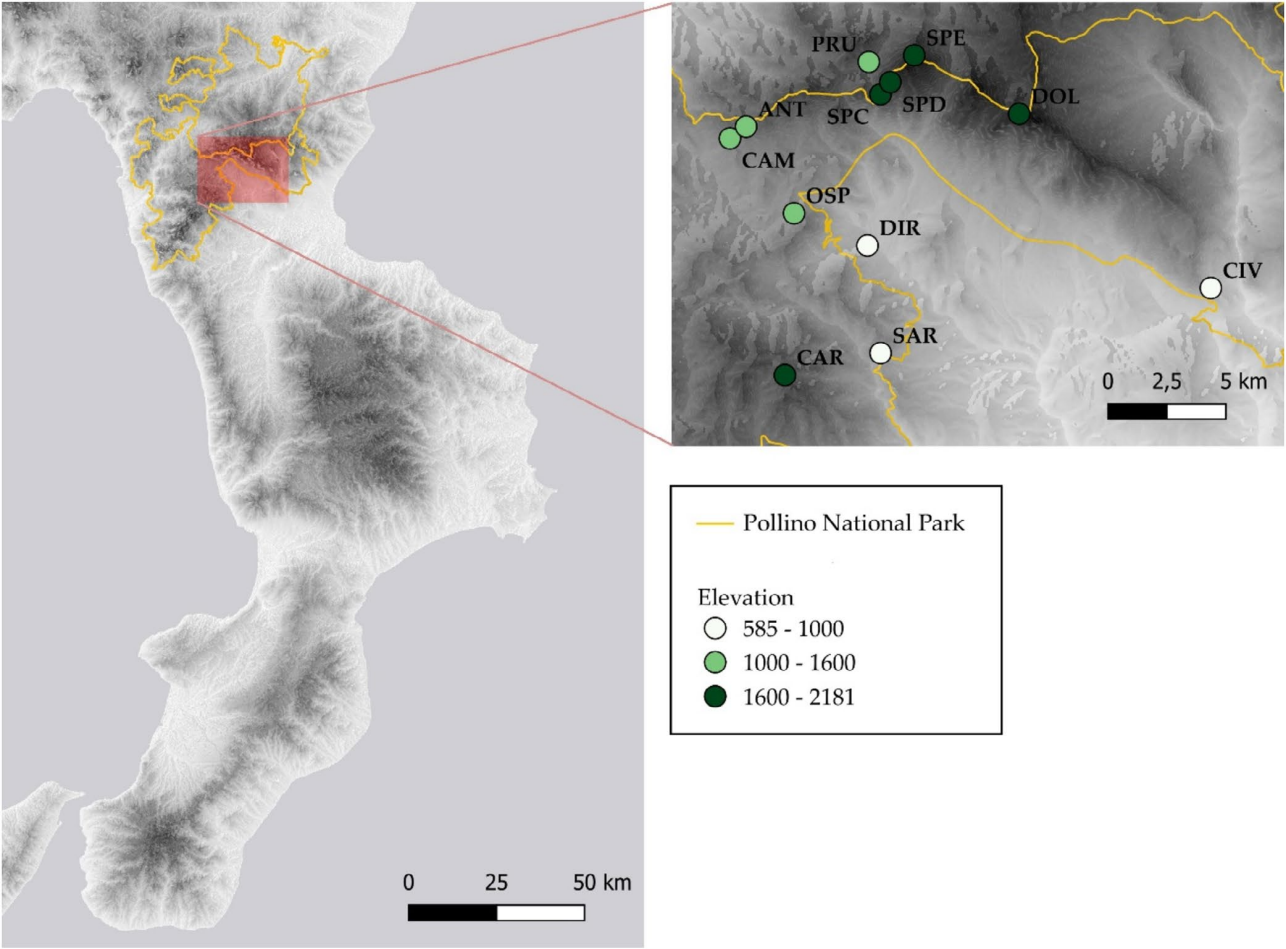
Distribution of the populations within the Pollino National Park, in the context of the Calabria region [background image from: Tinitaly (Tarquini et al. 2023)]. The legend indicates the grouping of populations into elevation categories.

For this study, we selected 12 populations of *D. virgineus* located within the Pollino National Park (Southern Italy) (Figure 1), a protected area occupying the southernmost portion of the Apennine range. The park covers a vast area (> 190,000 ha) and spans a wide altitudinal range, from just a few dozen meters above sea level to several mountain peaks exceeding 2100 m a.s.l. The sampled populations were distributed across an altitudinal gradient between 585 and 2181 m a.s.l. and were found in various types of calcareous rocky habitats (i.e., cliffs, pastures, and high‐altitude grasslands), in both partially wooded and open areas (Table 1). To facilitate the interpretation of bar charts, populations were categorized into three elevation classes: low (≤ 1000 m a.s.l.), intermediate (1000–1600 m a.s.l.), and high (> 1600 m a.s.l.) (Figure 1).

**TABLE 1:** List of the populations, sorted by elevation. The table includes the year in which the populations were sampled, geographic coordinates, elevation, habitat type (W = Woody; O = Open), number of sampled individuals, and the measured traits (FA, fluctuating asymmetry traits; Ft, functional traits; Mt, morphological traits).

Population	Acr.	Sampling year	Longitude (°)	Latitude (°)	Elevation (m a.s.l.)	Habitat	Sample size	Measure types
Morano	DIR	2015/2018	16°8′2.45”	39°50′50.51”	585	W	50	Mt‐Ft‐FA
Civita	CIV	2015	16°18′13.83”	39°49′45.98”	604	O	30	Mt
Saracena	SAR	2015	16°8′23.68”	39°48′22.64”	909	W/O	30	Mt
Ospedaletto	OSP	2018	16°5′52.65”	39°51′36.34”	1047	O	20	Mt‐Ft‐FA
Campotenese	CAM	2018	16°3′59.57”	39°53′19.64”	1194	W	14	Mt‐Ft‐FA
Anticristo	ANT	2018	16°4′28.77”	39°53′35.83”	1411	O	9	Mt‐Ft‐FA
Piano Ruggio	PRU	2018	16°8′9.27”	39°55′3.22”	1543	W/O	4	Mt‐Ft‐FA
Serra del Prete	SPC	2018	16°8′29.40”	39°54′18.37”	1727	O	20	Mt‐Ft‐FA
Caramolo	CAR	2015	16°5′32.73”	39°47′54.06”	1824	O	30	Mt
Serra del Prete	SPD	2018	16°8′47.69”	39°54′35.44”	1947	O	20	Mt‐Ft‐FA
Serra Dolcedorme	DOL	2015	16°12′35.47”	39°53′49.04”	2134	O	40	Mt
Serra del Prete	SPE	2015/2018	16°9′29.96”	39°55′10.82”	2181	O	30	Mt‐Ft‐FA

Data collection was conducted during two consecutive flowering seasons (i.e., 2015 and 2018), resulting in a total of 299 sampled individuals. Within each population, individuals were randomly selected, ensuring a minimum distance of 10 m between sampled plants to maintain data independence for statistical analyses.

Herbarium specimens were collected from individuals belonging to the populations DIR, OSP, CAM, ANT, PRU, and SPE, selected to collectively represent the diversity observed across the altitudinal gradient; the relative vouchers are stored at the Herbarium of the Botanic Garden of the University of Calabria (CLU).

All required authorizations for the sampling activities described above were provided by the Ente Parco Nazionale del Pollino as part of the agreement between the Ente Parco Nazionale del Pollino and the Department of Biology, Ecology and Earth Sciences of the University of Calabria, within the framework of the project ‘Monitoraggio delle specie della Flora di interesse conservazionistico presenti nei siti Natura 2000 del Versante Calabro del Parco Nazionale del Pollino’.

### 2.2 Morphological Traits

Morphological measures regarded both vegetative and reproductive traits, which have been emphasized for their taxonomic relevance in previous studies inherent in the *D. virgineus* group (Bacchetta et al. 2010; Brullo and Guarino 2017; Gargano et al. 2023). Overall, morphological measurements covered 18 phenotypic traits and involved 299 individuals from the 12 populations. However, due to the absence of certain organs in many individuals, the final morphological dataset used for subsequent analyses included 160 individuals and 13 traits (Table 2). These 13 traits comprised stalk height (height of the tallest floriferous scape), basal rosette area, length and width of both rosette and cauline leaves, patch size (number of flowering stalks per plant), number of flowers on the tallest stalk, calyx length and width, flower length, petal width, and corolla diameter.

**TABLE 2:** Complete list of the measured traits, distinguished in morphological, functional, and fluctuating asymmetry traits. Asterisks (*) indicate that the measurements were carried out on three pairs of cauline leaves (see Chapter 3.1).

Trait type	Trait name	Acronym	Unit
Morphological traits	Rosette area	Ra	cm^2^
	Rosette leaf length	RLl	mm
	Rosette leaf width	RLw	mm
	Cauline leaf length*	CLl	mm
	Cauline leaf width*	CLw	mm
	Stalk height	Sh	cm
	Patch size	Ps	count
	Flowers on stalk	Fn	count
	Flower length	FL	mm
	Calyx length	CAl	mm
	Calyx width	CAw	mm
	Petal width	PEw	mm
	Corolla diameter	COd	mm
Functional traits	Plant size	S	cm
	Leaf fresh mass	LfM	mg
	Leaf dry mass	LdM	mg
	Leaf area	LA	mm^2^
	Specific leaf area	SLA	mm^2^ *×* mg^−1^
	Leaf dry matter content	LDMC	%
Fluctuating asymmetry traits	Leaf length	Ll	mm
	Leaf perimeter	LP	mm
	Leaf area	LA	mm^2^
	Leaf fresh mass	LfM	mg
	Leaf dry mass	LdM	mg
	Specific leaf area	SLA	mm^2^ *×* mg^−1^
	Leaf dry matter content	LDMC	%

In populations CIV and SAR, plants were found in a late flowering stage, which prevented reliable measurement of flower length, petal width, and corolla diameter. Basal rosette area was estimated in the field by measuring two orthogonal diameters of the rosette and treating them as the axes of an ellipse to calculate the surface area. In the SPC population, plant conditions did not permit such measurements.

Measurements of cauline leaves were taken from three consecutive leaf pairs, starting from those closest to the flower. The first two leaves located immediately below the flower were excluded, as they were interpreted as floral bracts due to their morphology often differing from that of typical leaves.

### 2.3 Functional Traits

Functional analyses focused on six traits related to plant size and leaf characteristics (Table 2), with particular emphasis on leaf economics traits, which are efficient predictors of resource acquisition strategies and stress resistance. For example, high‐altitude plants often exhibit higher leaf dry matter content, thicker leaves, and lower specific leaf area and leaf nitrogen content, indicating a tendency toward a stress‐tolerant adaptation strategy (Cornelissen et al. 2003; Pérez‐Harguindeguy et al. 2013; Pierce et al. 2013).

Functional measurements were performed on 120 individuals (also included in the morphological dataset) of eight populations. These included one plant‐level trait, three directly measured leaf traits, and two derived leaf traits. Specifically, the six traits were: plant size (*S*), leaf fresh mass (LfM), leaf dry mass (LdM), leaf area (LA), specific leaf area (SLA = LA/LdM), and leaf dry matter content (LDMC = (LdM/LfM) × 100). Functional measurements were carried out following standardized protocols (Cornelissen et al. 2003; Pérez‐Harguindeguy et al. 2013). Unlike stalk height, which was included among the morphological traits, plant size (*S*) was defined as the shortest distance between ground level and the upper limit of the main photosynthetic tissues of the plant.

Plant‐level values of LfM, LdM, and LA were obtained by averaging measurements from four leaves per plant, including the two longest and best‐preserved basal rosette leaves and the second pair of cauline leaves. Healthy leaves were collected in the field and stored in nylon bags to prevent dehydration before laboratory processing. Each leaf underwent the following steps: (1) measurement of LfM using a digital precision balance (accuracy: 0.01 g); (2) taping onto a standard A4 paper sheet; (3) scanning using a flatbed scanner; (4) drying in an oven at 80°C for 72 h; and (5) measurement of LdM using an analytical balance (accuracy: 0.1 mg).

LA was calculated by processing the digital images with ImageJ software. To improve measurement accuracy, *S* and LA values were each taken twice by the same operator, and the two values were averaged to obtain the final score.

### 2.4 Fluctuating Asymmetry

The estimations of FAwere conducted on the same 120 individuals included in the functional dataset. FA is often interpreted as a proxy for developmental instability under environmental stress. These estimations comprised seven foliar traits (Table 2): leaf length (Ll), leaf perimeter (LP), LA, LfM, LdM, SLA, and LDMC. The latter five traits were assessed following the procedures previously described. In accordance with established protocols (Palmer and Strobecke 1986; Graham et al. 2010), FA was first calculated as the absolute difference between measurements of the two leaves at the same node. To account for differences in trait size and measurement scale, FA was then expressed as a proportional value by dividing the absolute difference by the value of the largest leaf within each pair: propFA = |*x*_1_ − *x*_2_|/max (*x*_1_, *x*_2_). This standardization allowed comparisons of asymmetry among traits measured in different units. Because no universal threshold exists for defining “high” FA or directly inferring developmental instability, proportional FA values were interpreted comparatively among populations and along the elevational gradient. For consistency, all measurements were performed on the second pair of cauline leaves for each sampled individual. Similar to LA, LP was determined by processing scanned leaf images using ImageJ. To enhance measurement accuracy, the measures for Ll, LP, and LA were repeated twice by the same operator, and the two values were averaged to obtain the final score.

### 2.5 Data Analysis

Data were organized into three distinct matrices corresponding to morphological, functional, and FA traits; the full dataset is provided inSupporting Information (Tables S1–, S3). As mentioned above, the morphological matrix (Table S1) comprised 160 individuals from 12 populations and 13 variables; the functional dataset (Table S2) included 120 individuals from eight populations and six variables; and the FA dataset (Table S3) consisted of 120 individuals from eight populations and seven variables.

Descriptive statistics were calculated for each trait, including the sample size, mean, 10th and 90th percentiles, and coefficient of variation (Table 3). Normality was assessed using the Shapiro–Wilk test on the original variables or, when necessary, on logarithmic transformed variables. The statistical analyses described below were performed in PAST and R (RStudio Team 2015) using the packages *dplyr*, *tidyr*, *VIM*, *naniar*, *mice*, *missForest*, *ade4*, *FactoMineR*, *factoextra*, *vegan*, and *permute*. For stochastic procedures, a fixed random seed (123) was used.

**TABLE 3:** Descriptive statistics on morphological, functional, and fluctuating asymmetry data. Number of individuals (*N*), mean, percentile 10th and 90th, coefficient of variation (CV), and significance of normality test (*p*) were reported. Asterisks (*) indicate that the variable was transformed before the normality test.

Trait	Trait type	*N*	Mean	10th percentile	90th percentile	CV	*p*
Ra	Morphological	129	166.356	32.98	345.51	91.372	0.474*
RLl	Morphological	160	8.582	3.2	14.1	84.874	—
RLw	Morphological	160	1.57	1.13	2.08	24.944	0.417*
CLl	Morphological	156	29.819	12	57	70.113	0.365*
CLw	Morphological	156	1.457	0.85	2.26	36.601	0.401*
Sh	Morphological	160	36.474	19.2	54.8	37.617	0.176*
Ps	Morphological	129	10.519	2	28	114.237	—
Fn	Morphological	160	2.094	1	4	54.31	—
FL	Morphological	119	23.024	19.66	27.06	12.671	0.105*
CAl	Morphological	160	19.644	16.56	22.74	12.568	0.531
CAw	Morphological	160	4.281	3.62	5.04	13.374	0.064*
PEw	Morphological	115	8.553	6.3	10.55	19.859	0.201
COd	Morphological	111	20.475	16.4	24.98	15.04	0.663
S	Functional	120	37.379	19.9	56.3	35.057	0.632
LfM	Functional	120	18.525	7.35	33.125	55.007	0.076
LdM	Functional	120	6.825	2.8	11.675	47.695	0.067
LA	Functional	120	63.74	29.991	103.98	46.925	0.114*
SLA	Functional	120	10.204	8.278	12.693	16.609	0.772
LDMC	Functional	120	40.665	31.9	50.902	18.805	0.078*
Ll	Fluctuating asymmetry	120	0.049	0.008	0.100	93.262	< 0.001
LP	Fluctuating asymmetry	120	0.051	0.011	0.092	80.287	< 0.001
LA	Fluctuating asymmetry	120	0.086	0.016	0.175	73.938	< 0.001
LfM	Fluctuating asymmetry	120	0.082	0.014	0.154	85.706	< 0.001
LdM	Fluctuating asymmetry	120	0.093	0.021	0.200	77.176	< 0.001
SLA	Fluctuating asymmetry	120	0.076	0.013	0.176	82.461	< 0.001
LDMC	Fluctuating asymmetry	119	0.059	0.009	0.132	90.634	< 0.001

#### 2.5.1 Univariate Analyses of Trait Variation

Logarithmic transformations were applied before univariate analysis, when necessary. Trait differences among populations were tested using one‐way ANOVA, when normal and homoskedastic distribution of residuals were fitted. When assumptions of homoscedasticity (Levene's test) were violated, Welch's ANOVA was applied. Post hoc comparisons were performed using Tukey's HSD. For not normally distributed residuals, the Kruskal–Wallis test and Dunn's test were used.

Relationships between traits and elevation were evaluated using linear regression models, with elevation treated as the independent variable. Model assumptions (linearity, normality, and homoscedasticity of residuals) were verified graphically (i.e., histograms, QQ‐plot, plots of standardized residuals vs. fitted values) and with Shapiro–Wilk and Breusch–Pagan tests. When extreme outliers were detected, their influence was evaluated, and analyses were repeated to assess the robustness of model assumptions.

#### 2.5.2 Ordination Analyses of Trait Variation

All continuous variables were standardized as *z*‐scores (*z* = [*x* − mean]/SD) before multivariate analyses. Patterns of morphological and functional variation among populations were analyzed by performing Principal Components Analysis (PCA) on morphological and functional datasets separately. Before ordination, collinearity among variables was assessed using Pearson's (*r*) or Spearman's (*ρ*) correlation coefficients, depending on data distribution. Variables showing |*r*| > 0.7 were excluded.

#### 2.5.3 Integration Between Morphological and Functional Trait Spaces

To assess congruence between morphological and functional trait spaces, three complementary analyses were performed on the imputed and standardized datasets. Imputed datasets were used to generate matrices for morphometric (X_morpho) and functional traits (X_func). PCA was conducted separately for the two data blocks (ade4::dudi.pca, centered and scaled; nf = 5). Then, the co‐structure between morphological and functional variation was assessed by co‐inertia analysis (ade4::coinertia), using the RV coefficient as a measure of association (0 = none, 1 = perfect). Significance was tested with 9999 permutations (RV.rtest).

Alignment of morphological and functional spaces was further evaluated with the *vegan* package, using Procrustes analysis and PROTEST (999 permutations). In addition, Mantel tests were applied to Euclidean distance matrices derived from morphological and functional trait spaces to assess the overall correlation between individuals in the two trait spaces. Permutation analyses were performed using population as a blocking factor to account for potential pseudoreplication.

#### 2.5.4 Multivariate Variance Partitioning

The relative contribution of environmental and functional predictors to morphological trait variation was quantified using multivariate variance partitioning based on redundancy analysis (RDA) (Legendre and Legendre 1998). All analyses were conducted on the subset of individuals for which complete information was available across morphological, functional, and environmental variables, resulting in a final dataset of 83 individuals (Table S4).

Before analysis, two morphological variables (patch size and rosette area) were excluded due to incomplete data and potential redundancy. The final morphological matrix included 11 traits, which were log‐transformed and standardized (mean = 0, SD = 1). Functional traits were summarized using the first two principal components (PC1 and PC2), representing the main axes of functional variation.

Environmental predictors included elevation (continuous variable) and habitat type (Open, Partially woody, Woody). Habitat type was coded as dummy variables, with Open treated as the reference category.

Variance partitioning was performed using adjusted *R* ^2^ values to quantify the proportion of morphological variance attributable to: (a) the pure environmental fraction, (b) the pure functional fraction, (c) the shared fraction between environmental and functional predictors, and (d) unexplained variance. In fraction (c), environmental predictors affect both functional and morphological traits, resulting in collinearity between environmental and functional predictors. Therefore, this fraction of explained variability should be attributed to environmental predictors.

Significance of the individual fractions was assessed using permutation tests (999 permutations), with permutations constrained within populations to account for potential nonindependence among individuals. The statistical significance test (*p*‐value) of the models and of the *R* ^2^ values was reported.

#### 2.5.5 Handling of Missing Data and Sensitivity Assessment

Patterns of missing data were quantified per trait and per population and visualized using summary tables and heatmaps (VIM::aggr and naniar::vis_miss). To evaluate the sensitivity of multivariate results to different treatments of missing data, three approaches were compared: (i) complete‐case analysis (excluding rows with missing values); (ii) multiple imputation using *mice* (*m* = 20, predictive mean matching for numeric variables); and (iii) random forest imputation using *missForest* (maxiter = 10, ntree = 500, variablewise = TRUE). Imputation accuracy was assessed using out‐of‐bag error (OOB) estimates per trait.

PCA was performed for each scenario (FactoMineR::PCA, scale. unit = TRUE). Population differences on PC1–PC2 were tested with ANOVA (or Welch's ANOVA when the assumption of homogeneity of variances was violated). Concordance between scenarios was evaluated with Procrustes analyses and PROTEST (*vegan*), and correlations of loadings for PC1 and PC2. Results were highly consistent across methods, suggesting that conclusions were not sensitive to the imputation strategy.

## 3 Results

### 3.1 Univariate Analyses of Trait Variation

#### 3.1.1 Morphological Traits

Most morphological variables exhibited a normal distribution, except for rosette leaf length, cauline leaf length, patch size, and number of flowers per stalk, which required logarithmic transformation to meet normality (Table 3). Correlation analyses revealed strong collinearity (*r* > 0.9) among length and width measurements of the three pairs of cauline leaves; therefore, only measurements from the second leaf pair were retained.

Vegetative traits generally showed broader dispersion, as indicated by higher coefficients of variation and the occurrence of several outlying values, whereas reproductive traits tended to show lower dispersion (Table 3). Most morphological traits differed significantly among populations (Table 4). Post hoc comparisons indicated that differentiation was primarily associated with populations located at the extreme limits of the studied altitudinal gradient, whereas intermediate‐elevation populations (e.g., ANT and PRU) displayed limited differentiation from both low‐ and high‐elevation populations (Table S5).

**TABLE 4:** Results of ANOVA/Kruskal–Wallis analyses testing differences among populations in morphological, functional, and fluctuating asymmetry data. Fisher value (*F*) and *H* value were reported together with the number of groups and of individuals and the significance test (*p*).

Trait	Trait type	Kruskal–Wallis	Welch ANOVA	ANOVA
Ra	Morphological	—	—	*F*(9,119) = 4.181, *p* < 0.001 (0.0001037)
RLl	Morphological	*H* (11) = 90.38, *p* < 0.0001	—	—
RLw	Morphological	—	*F*(11,45.4) = 9.07, *p* < 0.0001	—
CLl	Morphological	—	—	*F*(11,144) = 17.2, *p* < 0.0001
CLw	Morphological	—	*F*(11,41.9) = 28.68, *p* < 0.0001	—
Sh	Morphological	—	—	*F*(11,148) = 31.55, *p* < 0.0001
Ps	Morphological	*H* (9) = 43.889, *p* < 0.0001	—	—
Fn	Morphological	*H* (11) = 58.42, *p* < 0.0001	—	—
FL	Morphological	—	—	*F*(9,108) = 17.31, *p* < 0.0001
CAl	Morphological	—	—	*F*(11,148) = 22.43, *p* < 0.0001
CAw	Morphological	—	—	*F*(11,148) = 6.696, *p* < 0.0001
PEw	Morphological	—	—	*F*(9,104) = 10.12, *p* < 0.0001
COd	Morphological	—	—	*F*(9,100) = 2.507, *p* < 0.05 (0.01249)
S	Functional	—	—	*F*(7,112) = 41.19, *p* < 0.0001
LA	Functional	—	—	*F*(7,112) = 4.92, *p* < 0.0001
SLA	Functional	—	—	*F*(7,112) = 14.67, *p* < 0.0001
LDMC	Functional	—	—	*F*(7,109) = 41.56, *p* < 0.0001
Ll	Fluctuating asymmetry	*H* (7) = 10.57, *p* < 0.5	—	
LP	Fluctuating asymmetry	*H* (7) = 11.66, *p* < 0.5	—	
LA	Fluctuating asymmetry	*H* (7) = 19.04, *p* < 0.01	—	
LfM	Fluctuating asymmetry	*H* (7) = 13.47, *p* < 0.5	—	—
LdM	Fluctuating asymmetry	*H* (7) = 20.63, *p* < 0.01	—	—
SLA	Fluctuating asymmetry	*H* (7) = 12.66, *p* < 0.5	—	—
LDMC	Fluctuating asymmetry	*H* (7) = 13.70, *p* < 0.5	—	—

**FIGURE 2:**
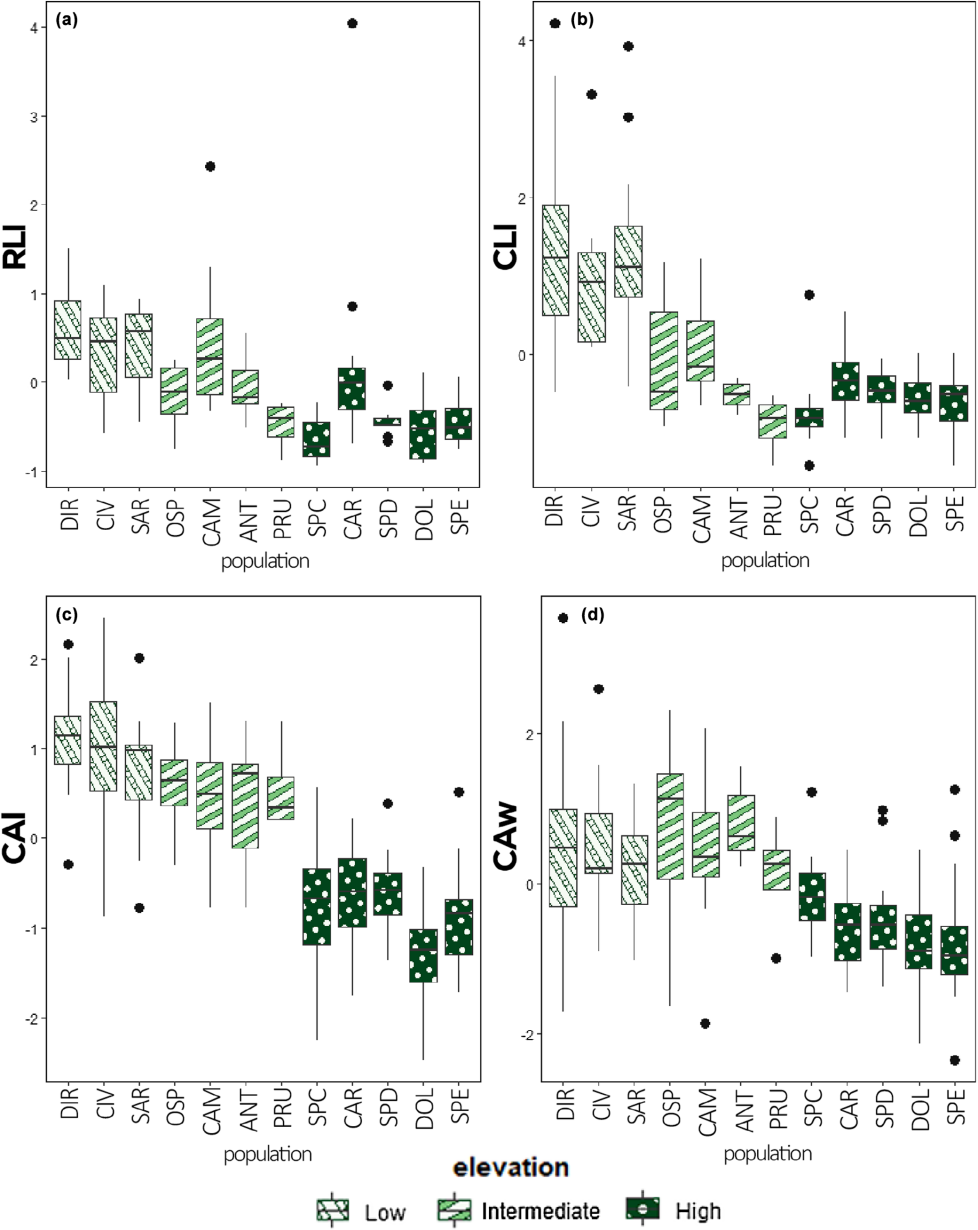
Boxplots showing the variation pattern of morphological traits among populations established from low‐ to high‐elevation (CAl, calyx length (a); CAw, calyx width (b); CLl, Cauline leaf length (c); RLl, Rosette leaf length (d)). The legend indicates the grouping of populations into elevation categories.

Some vegetative traits, such as rosette leaf length and cauline leaf width, exhibited pronounced differences between populations occurring below 1000 m a.s.l. (i.e., DIR, CIV, and SAR) and those at higher elevations (Figure 2; Table S5). However, these traits did not show significant variability among populations located at intermediate and higher elevations (e.g., ANT, PRU, and beyond) (Figure 2; Table S5).

Stalk height showed marked differences among populations below 1200 m a.s.l. and gradually decreased with increasing elevation (Table S5). Specifically, populations from partially woody habitats (e.g., DIR, SAR, and CAM) had taller stalks. However, the post hoc Tukey test indicated that stalk height variations among populations below 1200 m a.s.l. were generally not significant, except for CAM, which exhibited significantly taller stalks than other populations, and OSP, where stalks were notably shorter than those in DIR (Table S5). In contrast, populations at higher altitudes (e.g., SPD, DOL, and SPE) had significantly shorter stalks compared to those located below 1900 m a.s.l. (Table S5).

Overall, floral traits showed few population‐level differences below 1550 m a.s.l., according to post hoc comparisons (Table S5). However, these traits contributed to a marked differentiation between intermediate‐to‐low elevation populations and those above 1700 m a.s.l. Plants in high‐elevation populations had smaller calyces, both in length and width (Figure 2; Table S5). Especially, an altitudinal gradient in calyx size was evident. Populations at the lowest elevation (DIR and CIV at 500–600 m a.s.l.) displayed substantial variation in calyx size compared to populations at 1500 to 1700 m a.s.l., with a marked reduction observed in the uppermost populations (DOL, SPE above 2000 m a.s.l.).

Few morphological variables, including stalk height and rosette leaf length, required the removal of outliers to meet the assumptions of the regression analysis; while other traits, such as cauline leaf width and the number of flowers per stalk, failed to meet these assumptions and were excluded from the analyses. Several traits, including rosette leaf length, cauline leaf length, stalk height, calyx length, and calyx width, significantly decreased with altitude (Table 5). Notably, stalk height and calyx length exhibited the most pronounced reduction along the elevational gradient. Finally, some traits (e.g., patch size, rosette area) were not significantly related to elevation (Table 5).

**TABLE 5:** Results of regression tests assessing relationships between elevation and morphological, functional, and fluctuating asymmetry traits. For each model, the Fisher value (*F*), *p*‐value, and adjusted *R*‐squared (Adj *R* ^2^) are reported. The results of the Shapiro–Wilk test for normality of residuals and the Breusch‐Pagan test for homoscedasticity are also included.

Trait	Trait type	*F*	*p*	Adj *R* ^2^	Shapiro–Wilk	Breusch‐Pagan
Ra	Morphological	*F*(1,127) = 1.974	*p* < 1 (0.1625)	0.00755	*p* = 0.316	*p* = 0.1905
RLl	Morphological	*F*(1,155) = 101.8	*p* < 0.0001	0.3925	*p* = 0.456	*p* = 0.4026
RLw	Morphological	*F*(1,158) = 22.68	*p* < 0.0001	0.12	*p* = 0.6917	*p* = 0.08234
CLl	Morphological	*F*(1,154) = 53.35	*p* < 0.0001	0.3867	*p* = 0.2181	*p* = 0.1458
CLw	Morphological	*F*(1,154) = 6.939	*p* < 0.01 (0.009294)	0.0369	*p* = 0.01335	*p* = 0.0001
Sh	Morphological	*F*(1,158) = 118.6	*p* < 0.0001	0.4252	*p* = 0.06106	*p* = 0.9778
Ps	Morphological	*F*(1,127) = 0.9974	*p* < 1 (0.3198)	0.0001	*p* = 0.1132	*p* = 0.5316
Fn	Morphological	*F*(1,158) = 5.8	*p* < 1 (0.01718)	0.0293	*p* = 0.0003212	*p* = 0.6529
FL	Morphological	*F*(1,117) = 71.29	*p* < 0.0001	0.3733	*p* = 0.2214	*p* = 0.6717
CAl	Morphological	*F*(1,158) = 220	*p* < 0.0001	0.5794	*p* = 0.3623	*p* = 0.5405
CAw	Morphological	*F*(1,158) = 53.35	*p* < 0.0001	0.2477	*p* = 0.5165	*p* = 0.06481
PEw	Morphological	*F*(1,113) = 14.82	*p* < 0.001 (0.0001964)	0.1081	*p* = 0.3418	*p* = 0.7802
COd	Morphological	*F*(1,109) = 11.98	*p* < 0.001 (0.0007686)	0.09077	*p* = 0.1324	*p* = 0.765
S	Functional	*F*(1,118) = 117.3	*p* < 0.0001	0.4942	*p* = 0.7252	*p* = 0.7475
LA	Functional	*F*(1,118) = 10.59	*p* < 0.01 (0.001486)	0.07455	*p* = 0.445	*p* = 0.1614
SLA	Functional	*F*(1,118) = 11.49	*p* < 0.001 (0.0009498)	0.08104	*p* = 0.6851	*p* = 0.4577
LDMC	Functional	*F*(1,118) = 58.22	*p* < 0.0001	0.3284	*p* = 0.1106	*p* = 0.05746
Ll	Fluctuating asymmetry	*F*(1,114) = 0.452	*p* < 1	−0.0048	*p* < 0.001	*p* = 0.0713
LP	Fluctuating asymmetry	*F*(1,111) = 4.403	*p* < 0.05	0.0295	*p* < 0.001	*p* = 0.0681
LA	Fluctuating asymmetry	*F*(1,115) = 2.644	*p* < 1	0.0140	*p* < 0.0001	*p* = 0.147
LfM	Fluctuating asymmetry	*F*(1,113) = 3.643	*p* < 1	0.0227	*p* = 0.0109	*p* = 0.951
LdM	Fluctuating asymmetry	*F*(1,114) = 8.996	*p* < 0.01	0.0650	*p* = 0.0062	*p* = 0.0333
SLA	Fluctuating asymmetry	*F*(1,116) = 3.172	*p* < 0.1	0.0045	*p* < 0.001	*p* = 0.796
LDMC	Fluctuating asymmetry	*F*(1,113) = 0.000	*p* < 1	−0.0053	*p* < 0.001	*p* = 0.263

#### 3.1.2 Functional Traits

All functional traits were normally distributed and showed relatively narrow dispersion, as indicated by their coefficients of variation, with few outlying values (Table 3). Due to high multicollinearity with other variables, LfM and LdM were excluded from subsequent analyses (Table 6).

**TABLE 6:** Correlation matrix among functional traits. Above the diagonal are the *p*‐values (uncorrected), and below the diagonal are the Pearson correlation coefficients.

Trait	*S*	LfM	LdM	LA	SLA	LDMC
*S*	—	< 0.0001	< 0.0001	< 0.0001	0.0109	< 0.0001
LfM	0.5398	—	< 0.0001	< 0.0001	0.7351	< 0.0001
LdM	0.4164	0.9440	—	< 0.0001	0.0050	0.0376
LA	0.5467	0.9652	0.9224	—	0.4550	< 0.0001
SLA	0.2318	−0.0312	−0.2546	0.0688	—	< 0.0001
LDMC	−0.5120	−0.4259	−0.1900	−0.4065	−0.5465	—

**FIGURE 3:**
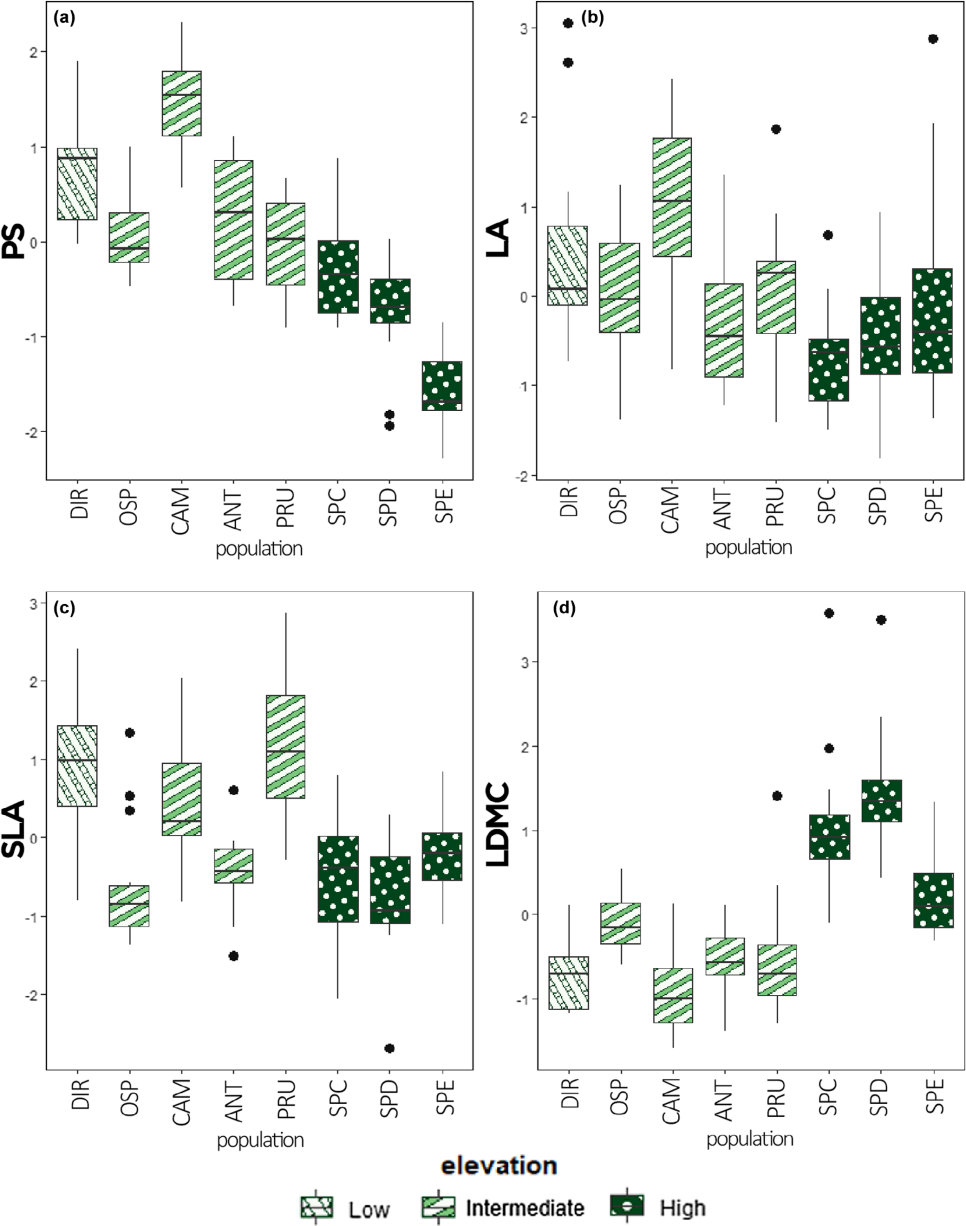
Boxplots showing the variation pattern of functional traits among populations established from low‐ to high‐ elevation (S, Plant size (a); LA, Leaf Area (b); SLA, Specific Leaf Area (c); LDMC, Leaf Dry Matter Content (d)). The legend indicates the grouping of populations into elevation categories.

Plant size (S) differed significantly among populations (Table 4), with CAM showing the highest values and SPE the lowest (Figure 3a and Table S6). Intermediate‐elevation populations (1000–1800 m a.s.l.) did not differ significantly (Table S6). LA also varied significantly (Table 4); however, post hoc Tukey tests (Table S6) identified significant differences only between CAM and ANT, SPC, and SPD, as well as between DIR and SPC. Similarly, although SLA exhibited significant variation (Table 4), no clear pattern associated with elevation emerged (Figure 3c). Specifically, DIR, CAM, and PRU displayed higher SLA values, while the remaining populations maintained lower values, irrespective of elevation (Figure 3). In contrast, LDMC varied significantly among populations (Table 4), with notably higher values in those above 1800 m a.s.l. (SPC, SPD, and SPE) (Table S6). More specifically, high‐elevation populations exhibited the highest LDMC values (Figure 3d), whereas populations at low to intermediate elevations maintained consistently low LDMC values and showed few differences according to post hoc comparisons (Table S6).

Regression analyses revealed significant relationships between elevation and S, LA, SLA, and LDMC (Table 5), with plant size and LDMC showing the strongest associations.

#### 3.1.3 Fluctuating Asymmetry

Proportional FA values varied significantly among populations only for LA and LdM (Tables 3 and 4). Dunn's post hoc tests with Bonferroni correction indicated that the significant pairwise contrasts mainly involved OSP, particularly against PRU and SPE for LA, and against CAM and SPE for LdM (Table S7). Along the elevational gradient, proportional FA generally tended to decrease, although significant negative relationships were detected only for LP and LdM (Table 5).

### 3.2 Ordination Analyses of Trait Variation

**FIGURE 4:**
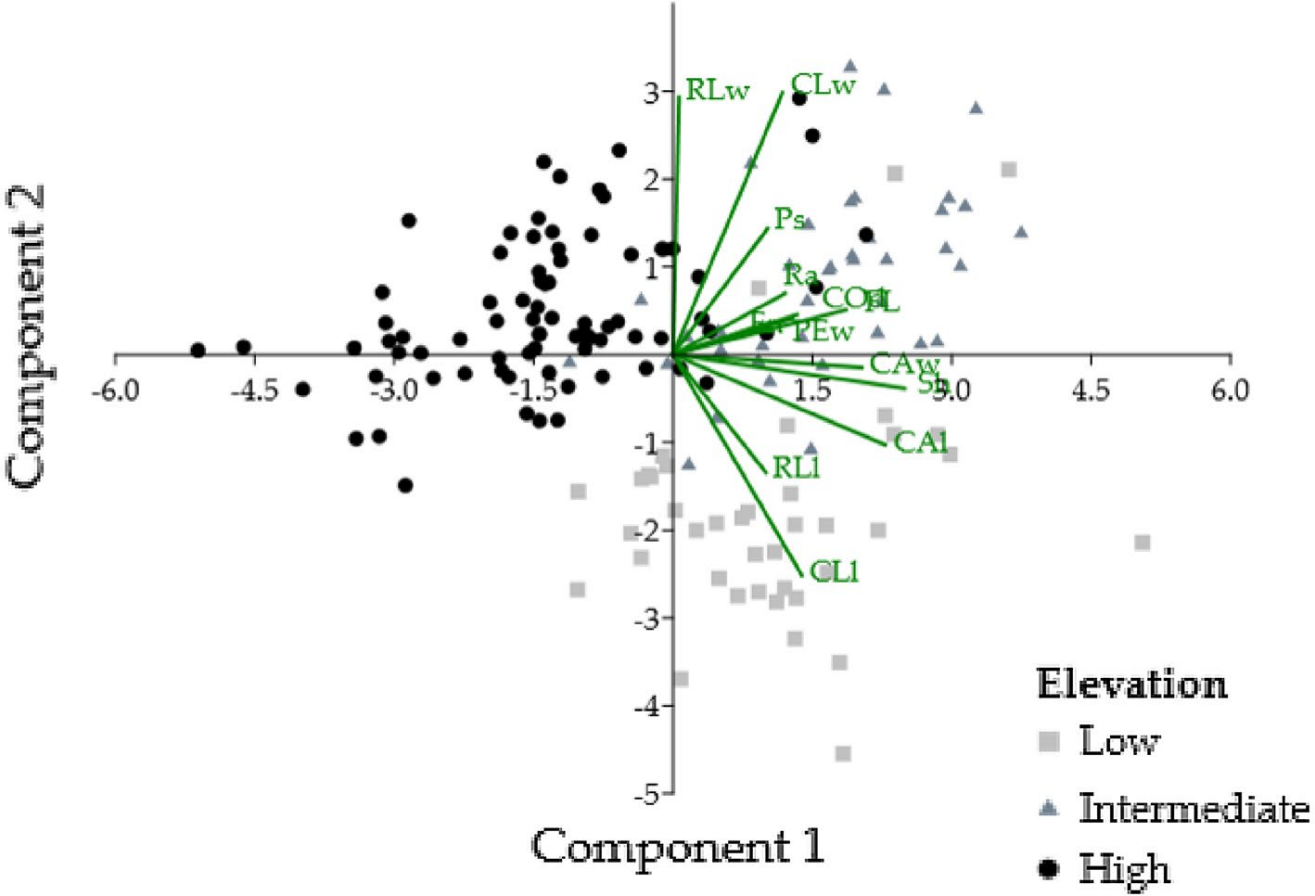
Biplot of the first two principal components of morphological traits, explaining for 28.865% and 17.758% of variance respectively. The legend indicates the grouping of populations into elevation categories.

The PCA on morphological traits confirmed that calyx length and width, stalk height, and rosette leaf length were the primary drivers of differentiation among low‐, mid‐, and high‐elevation populations (Figure 4). Separation along the first principal components (28.86% of variance) reflected the elevation gradient.

**FIGURE 5:**
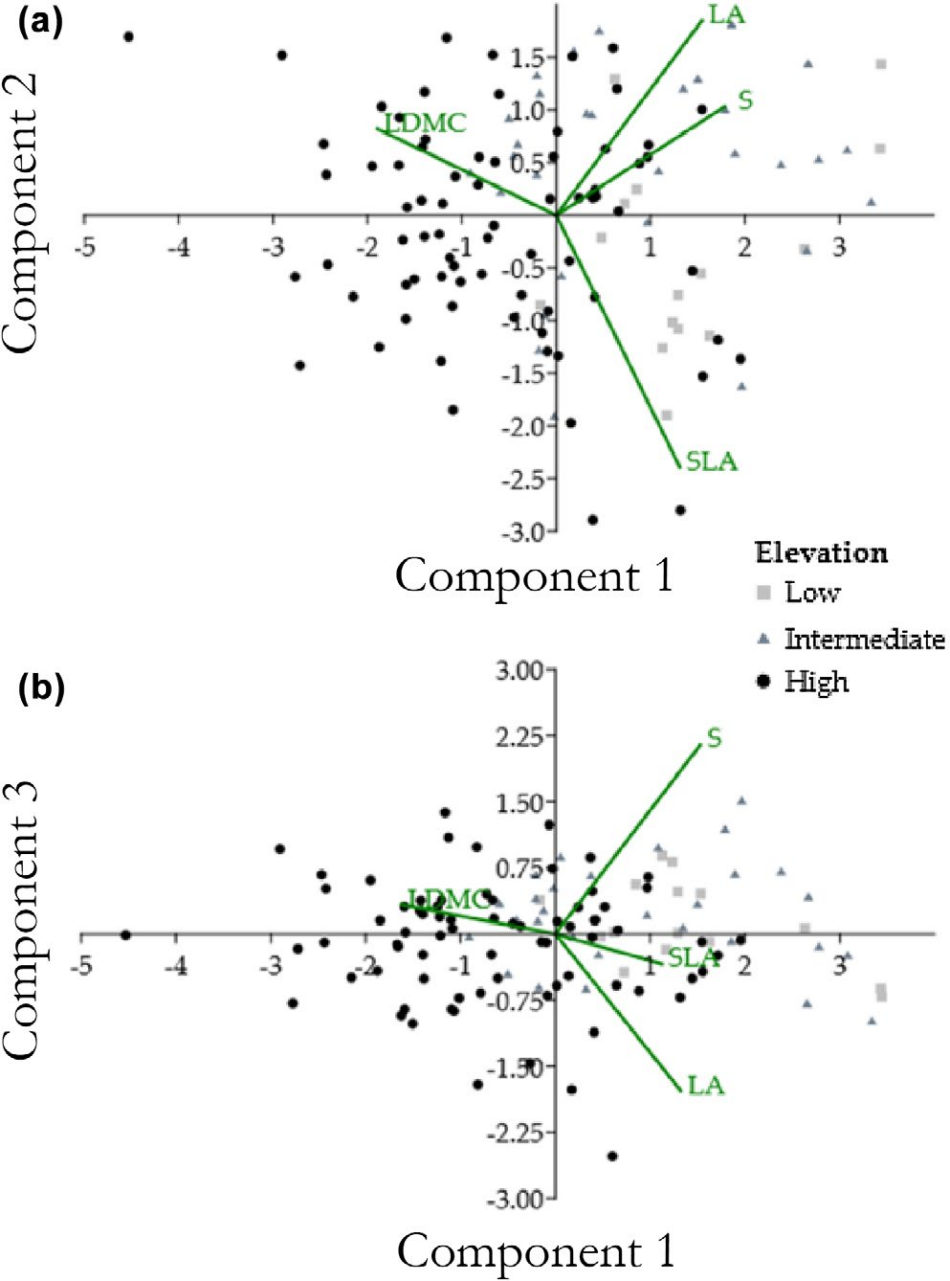
PCA on functional traits. The first three components make up for 54.722%, 25.868% (a) and 10.759% (b) of variance respectively. The legend indicates the grouping of populations into elevation categories.

The PCA performed on functional traits (Figure 5) further highlighted the influence of elevation. High LDMC values were characteristic of individuals sampled above 1800 m a.s.l., while individuals below 1500 m a.s.l. were characterized by larger S and higher LA and SLA values. Nevertheless, the two‐dimensional space defined by PC1–PC2 (54.72% of variance) (Figure 5a) and PC1–PC3 (25.87% of variance) (Figure 5b) did not provide a clear separation among populations. Most of the overlap in the biplots involved intermediate‐elevation populations (Figure 5a,b).

### 3.3 Integration Between Morphological and Functional Trait Spaces

Our analysis confirmed a significant congruence between patterns of morphological and functional trait variation. Co‐inertia analysis detected a significant covariation between morphological and functional trait matrices (RV coefficient = 0.408, *p* < 0.001; 9999 permutations), with the first co‐inertia axis explaining 95.5% of the total covariance. Accordingly, Procrustes analysis confirmed a significant congruence between the morphological and functional ordination spaces (Procrustes *r* = 0.50, *p* = 0.001). Finally, the Mantel tests pointed out a positive correlation between Euclidean distance matrices of morphological and functional data (*r* = 0.29, *p* < 0.0001).

### 3.4 Multivariate Variance Partitioning

Variance partitioning based on RDA showed that environmental and functional predictors jointly explained 32.0% of total morphological trait variation (adjusted *R* ^2^ = 0.320, *p* < 0.001).

Environmental predictors (elevation and habitat type) (*p* < 0.001) accounted for 28.6% of morphological variation when considered alone ([*a* + *c*]; Adj. *R* ^2^ = 0.286, *p* < 0.001); RDA1 was the only significant axis (*p* < 0.001), and elevation showed a major contribution to the axis.

Functional predictors (PC1 and PC2) accounted for 23.2% when considered alone ([b + c]; adjusted *R* ^2^ = 0.232, *p* < 0.0001), with RDA1 accounting for 94.4% of explained variation (*p* < 0.0001). However, when considering the effect of environmental predictors on functional predictors (*p* > 0.001), the former accounted for 35.2% of the explained variance, showing a significant effect of environmental predictors on functional traits.

Decomposition of the adjusted *R* ^2^ of the full model (*p* > 0.001) showed that the pure environmental fraction (a) (Env|Func_PCs) explained 8.9% of the total variation (adjusted *R* ^2^ = 0.0886, *p* < 0.001), the pure functional fraction (b) (Func_PCs|Env) accounted for 3.4% (adjusted *R* ^2^ = 0.0341, *p* < 0.001), and the shared fraction between environmental and functional predictors (c) accounted for 19.8% (adjusted *R* ^2^ = 0.1977). The remaining 67.96% of the variance corresponded to unexplained residual variation (d). Consequently, 28.6% of the explained morphological variability depended on environmental predictors and 3.5% on functional predictors.

Variance inflation factors ranged from 1.22 to 2.36, indicating no problematic multicollinearity among predictors.

### 3.5 Missing Data and Sensitivity Analyses

Missing values were heterogeneously distributed among traits and populations (Table S8; Figures S1–, S3). Sensitivity analyses demonstrated strong concordance among complete‐case, multiple imputation, and random forest approaches (Figures S4–, S6). PCA ordination spaces were highly similar across methods (Procrustes *r*≈0.93), and loadings correlations for PC1 and PC2 ranged between 0.87 and 0.89. Population differences along PC axes remained significant in all scenarios (*p* < 0.001; Table S8). Subsequent multivariate analyses were therefore based on the missForest‐imputed datasets.

## 4 Discussion

### 4.1 Patterns of Morphological Variation

Sensitivity analyses confirmed that the ordination framework was robust to missing data and consistently recovered a significant differentiation among populations along the main axes of variation, confirming the sensitivity of *Dianthus* phenotypes to elevation variation (Bacchetta et al. 2010; Castro et al. 2022; Franzoni, Astuti, Bartolucci, et al. 2024). Morphological divergence followed a clear elevation trend: low‐elevation populations comprised taller individuals with longer and narrower leaves, branched stems, more numerous flowers, and longer calyces, whereas high‐altitude populations were characterized by smaller plants with broader but shorter leaves, predominantly single stems, fewer flowers, and shorter calyces.

This trait combination corresponds closely to the diagnostic characters historically used to distinguish *D. longicaulis* and *D. brachycalyx* (Bacchetta et al. 2010). However, the limited differentiation of intermediate‐elevation populations and the absence of discrete morphological gaps indicate a clinal pattern of variation rather than discontinuous morphotypes. These results support previous doubts about the taxonomic consistency of the two entities (Franzoni, Astuti, Bacchetta, et al. 2024; Franzoni, Astuti, Bartolucci, et al. 2024).

This interpretation fits the broader morphometric pattern recently reported for the central Mediterranean *D. virgineus* complex, where current taxonomy was poorly supported and phenotypic variation was better described as a continuum of partially overlapping morphotypes, including the high‐elevation Apennine morphotype formerly referred to *D. brachycalyx* (Franzoni et al. 2025).

Overall, both vegetative and reproductive traits exhibited a general reduction with increasing elevation, consistent with the expectation that higher altitudes are associated with a reduction of the overall plant phenotype (Körner et al. 1989; Körner 2016). However, the relationships between the two trait types were not always linear. Some vegetative traits displayed substantial variability among populations at low to intermediate elevations. Especially, the populations occurring near forest edges (i.e., DIR, SAR, and CAM) significantly differed from others due to their larger stalks and leaves. This would represent a phenotypic response to microsite conditions that prevailed over general elevation‐driven parameters. As often recognized (Valladares et al. 2016), our data suggest such a local effect could be linked to the variation of ecological parameters driven by shading. Although the influence of local vegetation structure on morphological traits needs further investigation, it is evident that it contributes to increasing the difficulties in interpreting the overall variation pattern expressed by the study system.

On the contrary, the overall reduction in several reproductive traits (e.g., calyx and flower size, number of flowers) with increasing elevation appeared less influenced by local ecological factors. Although such a reduction can be driven by ecological restrictions due to abiotic parameters, it may also have implications for local patterns of plant‐pollinator interactions. For instance, given that *Dianthus* species are mainly butterfly‐pollinated (Erhardt 1990; Jürgens 2006), calyx length was demonstrated to play a key role in selecting their pollinators by matching the butterfly tongue length (Bloch et al. 2006; Bloch and Erhardt 2008). In such a pollination system, a longer calyx is expected to restrict the range of potential pollinators (Arroyo et al. 1982). Therefore, the shorter calyx observed in high‐altitude populations could allow a more generalized pollination strategy, potentially enhancing reproductive success under harsher conditions.

Overall, such outcomes indicate the trait combination traditionally used for taxonomic delimitation of *D. longicaulis* and *D. brachycalyx* includes a mixture of vegetative and reproductive characters that differ in their susceptibility to local ecological heterogeneity, as well as in their informative potential about the role of relevant ecological drivers promoting evolutionary divergence within this systematic group.

### 4.2 Pattern of Functional Variation and Relationships With Morphology

The functional traits examined in our study exhibited substantial variation along the analyzed elevational gradient. As frequently observed in studies assessing plant trait variation across elevational gradients (Di Biase et al. 2021; Stanisci et al. 2020), we found a significant reduction in plant size with increasing altitude, potentially related to shifts in life span, seed mass, and carbon storage capacity (Moles 2018).

The ordination analysis revealed opposing relationships between LDMC and SLA in response to elevation. Because these leaf economics traits represent contrasting strategies in regulating leaf photosynthetic efficiency and mechanical resistance, the observed increase in LDMC coupled with a reduction in SLA in high‐elevation populations suggests a shift toward a stress‐tolerant adaptive strategy (Díaz et al. 2015; Grime and Pierce 2012; Pierce et al. 2013). Overall, the described pattern of functional variation appears congruent with a gradual functional response to increasingly harsh climatic conditions (Grime and Pierce 2012; Körner 2016). Although our functional dataset focused on plant size and leaf traits, these variables represent widely used soft functional traits that summarize key axes of plant ecological strategies, particularly the size spectrum and the leaf economics spectrum. SLA, LDMC, and LA are commonly used as proxies for processes such as resource acquisition, structural investment, leaf longevity, and stress tolerance, which are more difficult to measure directly in large field‐based studies. Nevertheless, these traits do not exhaust the functional complexity of *Dianthus*, and future studies should integrate floral, phenological, reproductive, and pollinator‐related traits to obtain a broader functional characterization of the complex.

Importantly, the significant congruence detected between morphological and functional trait spaces indicates that these two sources of variation were not independent. The co‐inertia, Procrustes, Mantel, and variance partitioning analyses consistently showed that part of the morphological differentiation among populations was associated with functional variation. This suggests that the morphological characters historically used in taxonomic delimitation may also carry ecological‐functional information related to plant size and leaf economics.

At lower elevations, however, LDMC and SLA displayed less consistent patterns, suggesting that under less stressful conditions, different leaf traits may respond differently to local environmental variability or stochastic effects (Puglielli et al. 2024). Specifically, at low and intermediate elevations, we identified two population groups inhabiting different habitat types and displaying contrasting trait patterns: populations from shaded habitats (DIR, CAM, PRU) characterized by high SLA and low LDMC, and populations from open habitats (OSP, ANT) exhibiting low SLA and low LDMC. Then, in the latter case, reduced SLA likely reflects localized stress (e.g., higher solar radiation), which may limit investment in photosynthetic tissues without being sufficiently intense to promote a parallel increase in leaf structural consistency (as indicated by low LDMC). This confirms that LDMC is less responsive to fine environmental variations (Wilson et al. 1999; Roche et al. 2004). Therefore, as found for morphological traits, these habitat‐trait combinations recognized in low‐ to intermediate‐elevation sites could reflect functional responses to different ecological regimes (Grime and Pierce 2012; Wilson et al. 1999; Midolo et al. 2019), probably driven by small scale‐habitat heterogeneity that requires further work to be fully disentangled.

The ecological relevance of observed morphological patterns is also supported by the variation in proportional FA along the elevational gradient. Contrary to the expectation of increased developmental instability under stressful environmental conditions (e.g., Palmer and Strobecke 1986; Palmer 1994; Graham 2021), proportional FA did not increase toward higher elevations. Rather, selected FA traits showed significant negative relationships with elevation, suggesting a tendency toward reduced asymmetry in high‐elevation populations. Conversely, the higher proportional FA observed in some low‐ to intermediate‐elevation populations may reflect greater developmental lability under more heterogeneous local conditions, potentially contributing to the broader morphological and functional variability observed in these populations.

However, this pattern was trait‐specific and should therefore be interpreted cautiously. In effect, while extreme environments are commonly associated with increased stress and plastic phenotypic responses, persistent stress may also favor enhanced developmental stability (West‐Eberhard 1989; Visser et al. 2003). The reduced proportional FA observed for some traits in high‐elevation conditions may therefore reflect enhanced developmental buffering under recurrent environmental constraints, possibly involving phenotypic or environmental canalization (Debat and David 2001; Siegal and Bergman 2002; Klingenberg 2019; Takahashi 2019). In this perspective, the functional shift toward stress tolerance, mainly expressed by increased LDMC and reduced SLA, together with the tendency toward reduced FA in selected traits, is consistent with a scenario of environmentally mediated differentiation, potentially involving local adaptation. Furthermore, the recurrence of parallel phenotypic patterns across independent mountain systems (Franzoni et al. 2023; Franzoni, Astuti, Bartolucci, et al. 2024) further suggests that this mechanism may operate at broader spatial scales within the genus.

Overall, different from other study cases in the genus (Rocha et al. 2026), our integrative analytical framework indicated the occurrence of significant associations among morphological patterns, functional traits, and elevation, suggesting that the morphological variability characterizing the studied populations of *D. virgineus* has a strong ecological basis.

However, elevation should be interpreted here as a proxy for multiple environmental drivers rather than as a causal factor per se.

Because our sampling is restricted to a southern Apennine elevational gradient, these morpho‐functional–environmental associations should be interpreted as a local case study within the broader *D. virgineus* complex. Future analyses integrating additional populations, mountain systems, climatic contexts, and fine‐scale edaphic and microhabitat variables will be necessary to identify the specific environmental factors underlying these patterns and to test whether similar functional and developmental responses recur across the complex. Together with the persistence of such differences in common garden conditions (D.G., pers. obs.), these findings support a possible adaptive component of the observed phenotypic shifts, potentially reflecting local selection despite ongoing gene flow within the whole metapopulation system (Franzoni et al. 2023). Nevertheless, because our design does not directly separate phenotypic plasticity from genetically based local adaptation, this interpretation should be considered as a plausible evolutionary scenario rather than a definitive demonstration. From a systematic standpoint, such environmentally associated morphological divergence coupled with maintained gene flow may generate mismatches between genetic and phenotypic patterns (Franzoni et al. 2023), complicating the taxonomic interpretation of differentiated morphotypes.

## 5 Conclusions

Our study evidenced significant morphological and functional variability in *D. virgineus* along a wide elevational gradient in the Southern Apennines. The overall pattern indicates the occurrence of continuous variation, with no evident discontinuity justifying the taxonomic separation of low‐ and high‐elevation morphotypes and aligning with recent genetic studies on the *D. virgineus* complex. Nonetheless, our data also indicated a strong congruence between morphological and functional traits, suggesting that a substantial fraction of observed morphological variation may be shaped by environmental constraints. Functional traits such as SLA and LDMC showed contrasting responses to elevation, with patterns consistent with a shift toward a stress‐tolerant strategy at higher elevations. At lower elevation, trait variability appeared more context‐dependent, likely shaped by microhabitat heterogeneity and minor ecological constraints. Accordingly, the tendency toward reduced proportional FA in selected traits at higher elevations suggests enhanced developmental stability, likely promoted by environmental canalization under persistent environmental constraints. Such findings support the idea that morphotypes of *D. virgineus* occurring at different elevations may include locally adapted phenotypes components, while taxonomic cohesion is preserved by ongoing gene flow.

Our work also emphasizes that integrating traditional morphological data, measures of functional traits, and genetic information may represent a useful way for understanding the ecological and evolutionary value of phenotypic variation in polymorphic systematic lineages, thus allowing more consistent taxonomic interpretation.

## Author Contributions

**Simone Rovito:** conceptualization (equal), data curation (equal), formal analysis (equal), investigation (equal), writing – original draft (equal). **Domenico Amantea:** data curation (equal), formal analysis (equal), writing – original draft (equal). **Nicodemo Giuseppe Passalacqua:** formal analysis (equal), investigation (equal), methodology (equal), writing – review and editing (equal). **Liliana Bernardo:** investigation (equal), methodology (equal), writing – review and editing (equal). **Domenico Gargano:** conceptualization (lead), formal analysis (equal), methodology (equal), supervision (equal), validation (equal), writing – review and editing (lead).

## Funding

The authors have nothing to report.

## Conflicts of Interest

The authors declare no conflicts of interest.

## Supporting information


**Figure S1:** Visualization of the distribution and patterns of missing data across the sampled populations and traits.

**Figure S2:** Additional visualizations detailing missing data patterns and combinations across the dataset.

**Figure S3:** Heat map showing percentage (%) of missing data per trait.

**Figure S4:** Principal component analysis (PCA) ordination biplot based on the complete‐case dataset. The legend indicates the grouping of populations into elevation categories.

**Figure S5:** Principal component analysis (PCA) ordination biplot based on the dataset imputed via multiple imputation (*mice*), generated for the sensitivity analysis of missing data treatments.

**Figure S6:** Principal component analysis (PCA) ordination biplot based on the dataset imputed via random forest (*missForest*), generated for the sensitivity analysis of missing data treatments.

**Table S1:** Full morphological dataset. The matrix comprises 13 morphological variables (vegetative and reproductive traits) measured on 160 individuals of *Dianthus virgineus* sampled across 12 populations.

**Table S2:** Full functional traits dataset. The matrix comprises six functional traits related to plant size and leaf characteristics measured on 120 individuals of *Dianthus virgineus* sampled across eight populations.

**Table S3:** Full fluctuating asymmetry (FA) dataset. The matrix consists of FA estimations for seven foliar traits measured on 120 individuals of Dianthus virgineus sampled across eight populations.

**Table S4:** Complete integrated dataset used for multivariate variance partitioning (Redundancy Analysis, RDA). The subset includes 83 individuals and 11 morphological traits.

**Table S5:** Between‐population post hoc tests relative to vegetative and sexual morphological traits. For the variables RLi, Ps, and NF the table shows the *p*‐value produced by the Dunn post hoc test; for all other variables, it is provided the value of (i‐j) under the Tukey post hoc test. The elevation of each population appears in parenthesis. Legend for symbols: *, the test is significant at the 0.05 level; **, the test is signifcant at the 0.01 level; −, data not available to perform the test. Legend for vegetative traits: CLl, Cauline leaf length; CLw, Cauline leaf width; Ps, patch size; Ra, Rosette area; RLl, Rosette leaf length; RLw, Rosette leaf width; Sh, stalk heigth. Legend for sexual traits: CAl, calyx length; CAw, calyx width; COd, corolla diameter; Fl, flower length; NF, no. of flowers on the stalk; PEw, petal width. Significant differences are evidenced in light gray.

**Table S6:** Between‐population post hoc tests relative to functional traits. For all variables, it is provided the value of (i‐j) under the Tukey post hoc test. The elevation of each population appears in parenthesis. Legend for symbols: *, the test is significant at the 0.05 level; **, the test is signifcant at the 0.01 level; −, data not available to perform the test. Legend for variables: LA, leaf area; LDMC, leaf dry matter content; LDW, leaf dry weight; LFW, leaf fresh weight; Ps, plant size; SLA, specific leaf area. Significant differences are evidenced in light gray.

**Table S7:** Between‐population post hoc tests relative to fluctuating asymmetry traits. For the variables SLA, LDMC, LFW, and LDW, the table shows the *p*‐value produced by the Dunn post hoc test; for all other variables, it is provided the value of (i‐j) under the Tukey post hoc test. The elevation of each population appears in parenthesis. Legend for symbols: *, the test is significant at the 0.05 level; **, the test is signifcant at the 0.01 level; −, data not available to perform the test. Legend for variables: LA, leaf area; LDMC, leaf dry matter content; LDW, leaf dry weight; LFW, leaf fresh weight; Ll, leaf length; Lp, leaf perimeter; SLA, specific leaf area. Significant differences are evidenced in light gray.

**Table S8:** results of ANOVA evaluating population differences along the first two principal components (PC1 and PC2) across the three missing data handling scenarios (complete‐case, *mice*, and *missForest*).

## Data Availability

All research data that the paper refers to are provided as Supporting Information. Field activities, including the collection of plant material, were authorized by Ente Parco Nazionale del Pollino within the framework of the project ‘Monitoraggio delle specie della Flora di interesse conservazionistico presenti nei siti Natura 2000 del Versante Calabro del Parco Nazionale del Pollino’. All voucher specimens are deposited within the CLU herbarium under accession numbers 26306–26311 and are available from the authors upon request.
