# Supplementary figures and images for: Elevation‐Driven Morphological Variation in Dianthus virgineus L. s.l. in the Southern Apennine: A Functional Perspective
Source: Ecol Evol. 2026 Jul 8;16(7):e73898. doi: 10.1002/ece3.73898 (PMC13345778; doi:10.1002/ece3.73898)

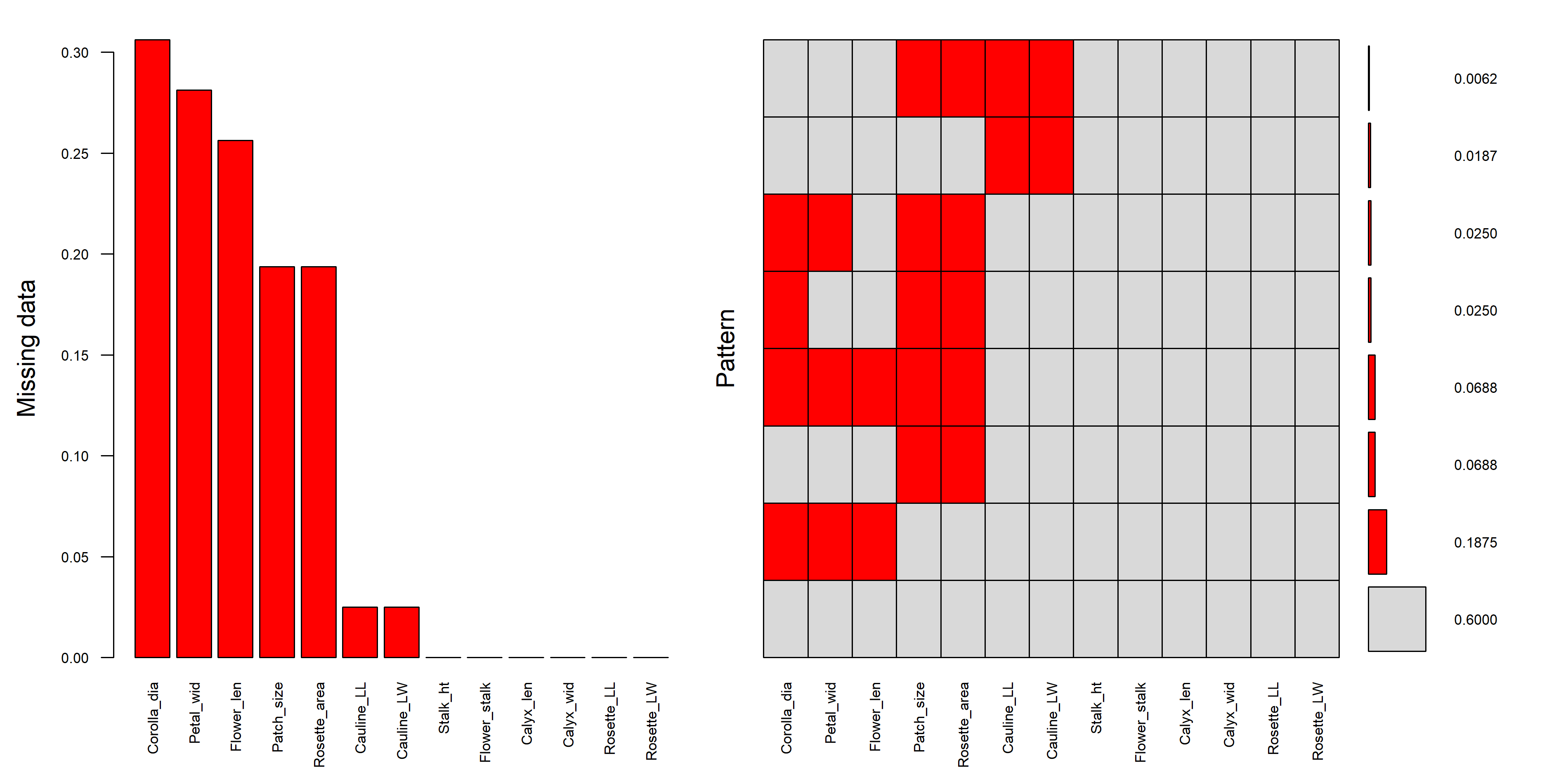

Supplement: Supplementary file 1 — Figure S1: Visualization of the distribution and patterns of missing data across the sampled populations and traits. [file ECE3-16-e73898-s004.png]

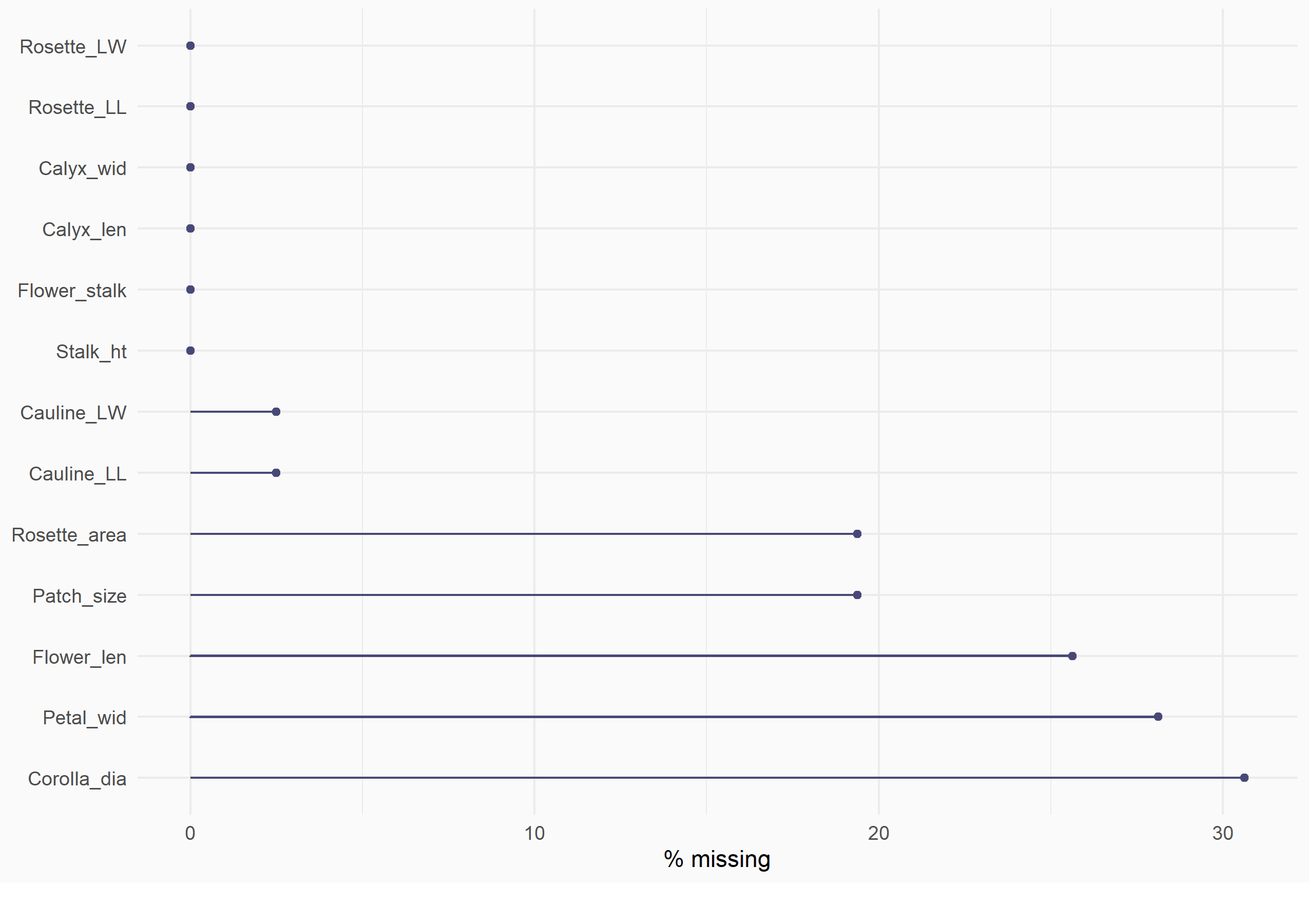

Supplement: Supplementary file 2 — Figure S2: Additional visualizations detailing missing data patterns and combinations across the dataset. [file ECE3-16-e73898-s005.png]

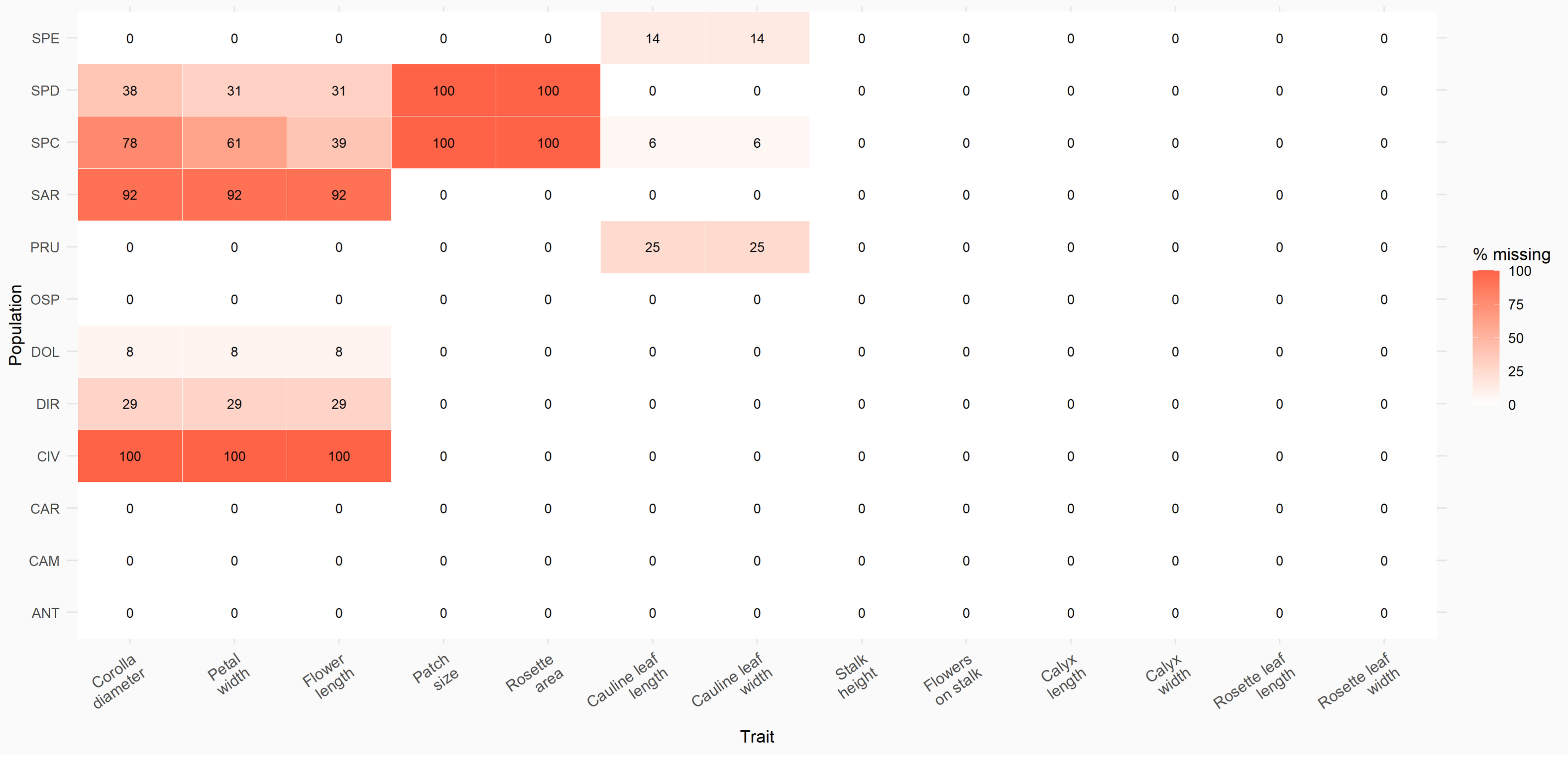

Supplement: Supplementary file 3 — Figure S3: Heat map showing percentage (%) of missing data per trait. [file ECE3-16-e73898-s008.jpg]

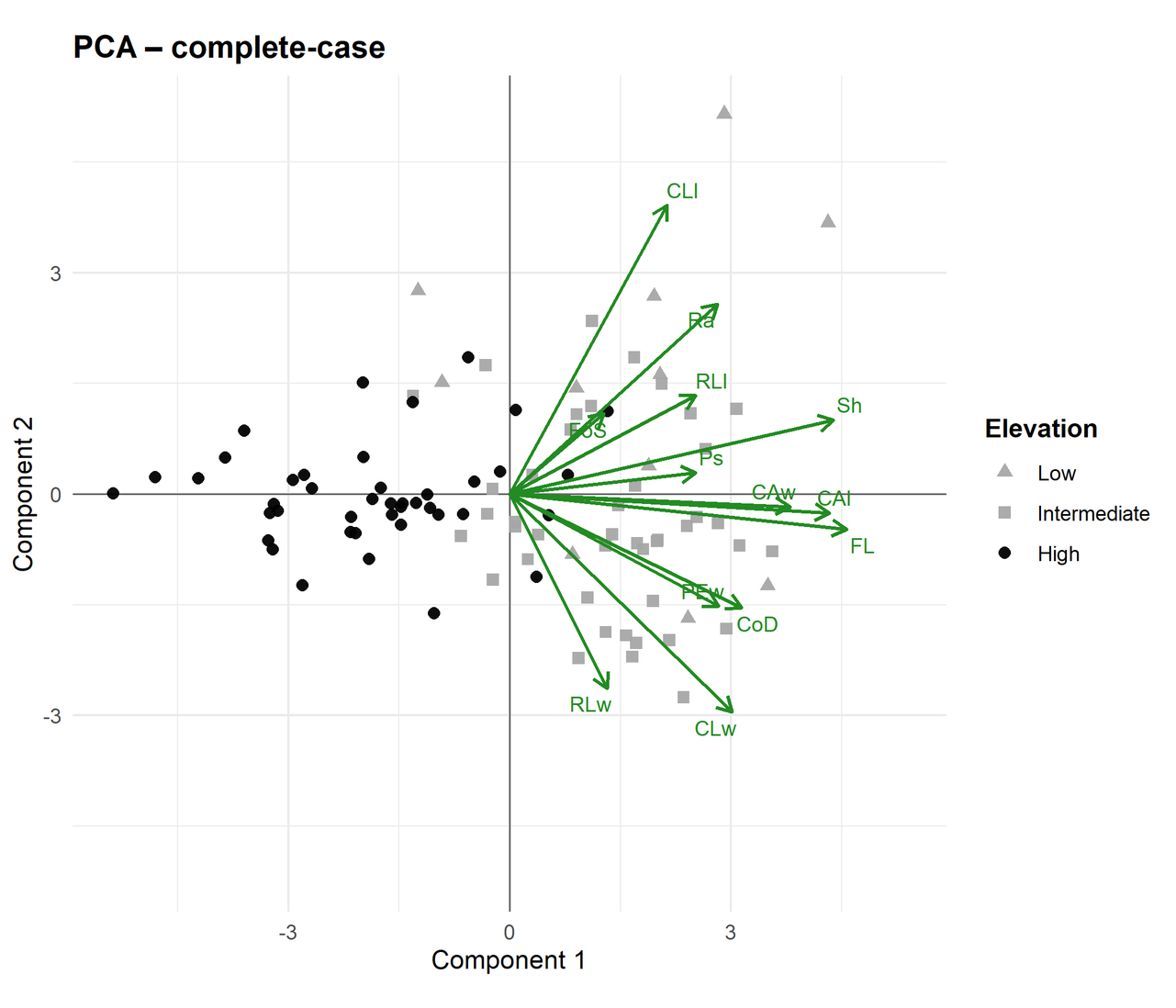

Supplement: Supplementary file 4 — Figure S4: Principal component analysis (PCA) ordination biplot based on the complete‐case dataset. The legend indicates the grouping of populations into elevation categories. [file ECE3-16-e73898-s007.jpg]

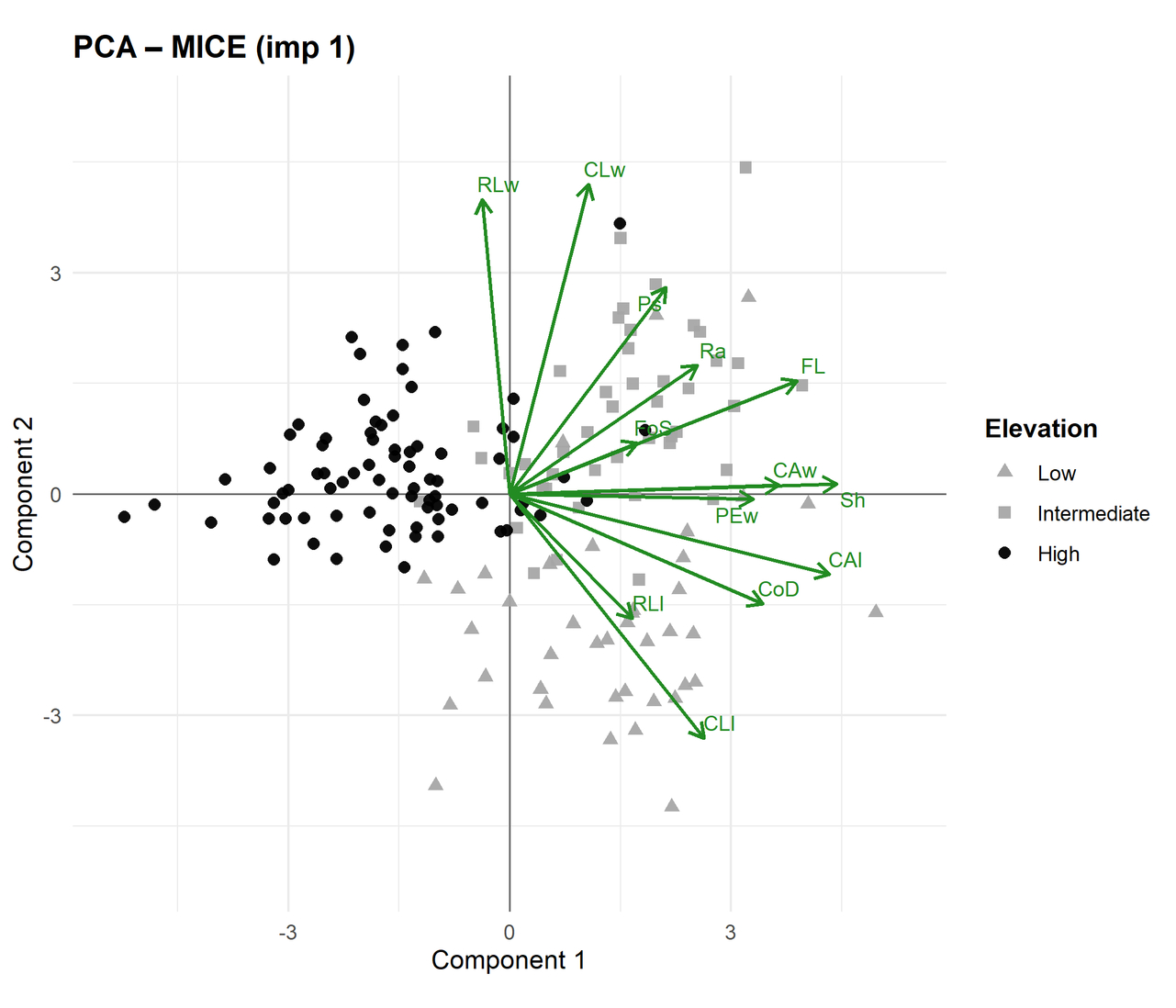

Supplement: Supplementary file 5 — Figure S5: Principal component analysis (PCA) ordination biplot based on the dataset imputed via multiple imputation (mice), generated for the sensitivity analysis of missing data treatments. [file ECE3-16-e73898-s014.jpg]

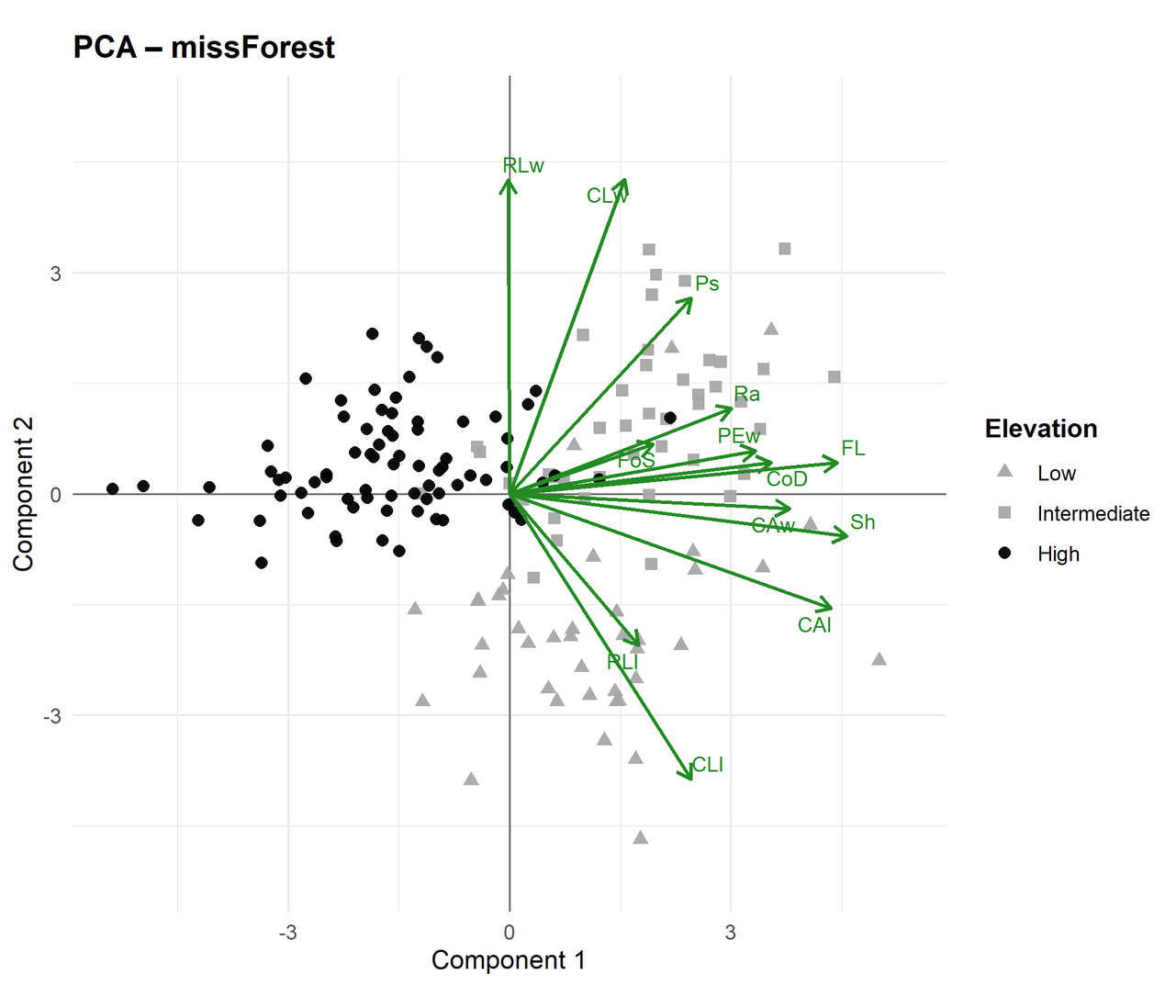

Supplement: Supplementary file 6 — Figure S6: Principal component analysis (PCA) ordination biplot based on the dataset imputed via random forest (missForest), generated for the sensitivity analysis of missing data treatments. [file ECE3-16-e73898-s010.jpg]
